# Acclimation to high and low diurnal light is flexible in *Chlamydomonas reinhardtii*

**DOI:** 10.1073/pnas.2523996123

**Published:** 2026-01-02

**Authors:** Sunnyjoy Dupuis, Jordan L. Chastain, Genevieve Han, Victor Zhong, Sean D. Gallaher, Carrie D. Nicora, Samuel O. Purvine, Mary S. Lipton, Krishna K. Niyogi, Masakazu Iwai, Sabeeha S. Merchant

**Affiliations:** ^a^California Institute for Quantitative Biosciences, University of California, Berkeley, CA 94720; ^b^Department of Plant and Microbial Biology, University of California, Berkeley, CA 94720; ^c^Molecular Biophysics and Integrated Bioimaging Division, Lawrence Berkeley National Laboratory, Berkeley, CA 94720; ^d^Department of Molecular and Cell Biology, University of California, Berkeley, CA 94720; ^e^Earth and Biological Sciences Division, Pacific Northwest National Laboratory, Richland, WA 99352; ^f^HHMI, University of California, Berkeley, CA 94720; ^g^Innovative Genomics Institute, University of California, Berkeley, CA 94720; ^h^Environmental Genomics and Systems Biology Division, Lawrence Berkeley National Laboratory, Berkeley, CA 94720

**Keywords:** diel, photoprotection, NPQ, chloroplast swelling, heat shock response

## Abstract

Chlamydomonas acclimates to repeated low (LL) or high light (HL) days by changing the abundance of photosynthetic complexes and the ultrastructure of its thylakoid membranes. These phenotypes persist through the night phases, suggesting a readiness for the daylight environment that is routinely experienced despite the intervening dark periods [S. Dupuis *et al.*, *Plant Cell* **37**, koaf086 (2025), 10.1093/plcell/koaf086]. Here, we investigate how prior acclimation impacts algal fitness upon a change in daylight intensity and how quickly Chlamydomonas can reprogram its photoprotective strategy in a diurnal context. We performed a systems analysis of synchronized populations acclimated to diurnal LL when subjected to HL days and of populations acclimated to diurnal HL when subjected to LL days. In the latter case, diurnal photoacclimation decreased fitness during the first day at a new light intensity: HL-acclimated cells barely increased in size over the first LL period, and they failed to complete a cell cycle. However, although LL-acclimated cells showed severe photodamage after 6 h of HL, they recovered chloroplast form and function later that afternoon and successfully divided at nightfall. These cells rapidly altered their thylakoid membrane ultrastructure, increased their photoprotective quenching capacity, and decreased their inventory of photosystem and antenna proteins by the end of the first HL day. Transcriptomic and proteomic analyses revealed rapid induction of thousands of genes, including those encoding proteases, chaperones, and other proteins involved in the chloroplast unfolded protein response. These results show that the alga is highly flexible and competent to rapidly acclimate to changes in diurnal light intensity.

## Significance

Photosynthetic microbes are major contributors to the global carbon cycle, and they have strategies to succeed in the dynamic light environment of our planet. When it experiences repeated limiting or excess light days, the model alga *Chlamydomonas reinhardtii* maintains optimal thylakoid membrane architecture, photosynthetic complexes, and photoprotective capacity for the routine light intensity. Here, we investigate how maintenance of these phenotypes into the night impacts algal fitness upon a change in daylight intensity. We find that cells accustomed to dim days experience severe photoinhibition during a surprising bright day and their chloroplast integrity suffers. Nevertheless, they grow, divide, and flexibly adjust their photoprotective strategy in just 1 d. We chart the transcriptome and proteome as Chlamydomonas acclimates anew.

Algae are beholden to the Sun and the dynamic light environments found on our planet. As the Sun rises, sets, and is blocked by clouds or other obstructions, the incident light intensity can vary by several orders of magnitude (1–3). Algae must absorb the incident light with light-harvesting pigment–protein complexes and direct the energy to the reaction centers of their photosystems to generate chemical energy for carbon assimilation and growth. Yet, absorbing more light energy than can be processed by the slower downstream reactions can cause photooxidative damage and decrease photosynthetic efficiency.

To succeed in dynamic light environments, photosynthetic microbes modulate the amount of light they absorb, dissipate excess light as heat through nonphotochemical quenching (NPQ), and repair photooxidative damage. While NPQ mechanisms can be activated and relaxed within minutes (4, 5), changes in chlorophyll (Chl) content and light-harvesting antenna size occur only after hours of exposure to high light (HL) or low light (LL), a process called photoacclimation (6–9). Upon prolonged HL, the popular reference alga *Chlamydomonas reinhardtii* (Chlamydomonas hereafter) decreases its cellular Chl content and light-harvesting complex (LHC) abundance to reduce the excitation energy pressure on the photosystems (10–15). In addition, the thylakoid membranes that house the LHCs and photosystems undergo changes in surface area and stacking, altering the interactions between the photosynthetic electron transfer chain complexes and other proteins, including those important for photosystem II (PSII) repair (6, 16, 17).

Prolonged HL exposure also leads to accumulation of the stress-related LHC-like proteins LHCSR3 and LHCSR1, which perform energy-dependent NPQ (qE) in Chlamydomonas (18–20). Upon acidification of the thylakoid lumen in excess light, these Chl- and carotenoid-binding proteins undergo rapid, pH-dependent conformational changes to turn into strong quenchers of excited Chl (19, 21–25). Expression of *LHCSR* and *PSBS* transcripts is suppressed in the absence of excess light or other activating signals (3, 26–29), as even their unprotonated protein products can quench excited Chl and decrease photosynthetic efficiency in LL (22). Yet, LHCSR proteins exhibit half-lives of 20 to 30 h in HL-acclimated Chlamydomonas cells transitioned to LL, persisting over the diurnal cycle and leading to constitutive NPQ capacity (30–32).

Several studies have investigated the kinetics of photoacclimation in Chlamydomonas transitioned between continuous LL and HL (11, 13, 33–36). These studies suggest that the alga can begin decreasing cellular Chl content, LHC abundance, and thylakoid membrane stacking within just 1 to 2 h of increased irradiance. However, the diurnal light cycles of our natural environment lead to rhythmic growth, metabolism, and gene expression, which could impact acclimation competency at a given time of day. In addition, the periodic darkness of night may interrupt acclimation processes and delay changes in cellular Chl content (37).

Chlamydomonas is an excellent reference organism for studying diurnal behavior. Like other photosynthetic organisms, its growth and metabolism are coordinated with time of day thanks to the periodic availability of light, which serves as both an energy source and a signal (38). As a result, Chlamydomonas populations synchronize when grown under repeated diurnal cycles in the laboratory: cells progress through the G1 phase during the daytime, undergo multiple fission S/M cycles at dusk to produce daughter cells of equal sizes, and then remain in a quiescent G0 phase of the cell cycle during the night. Synchronous populations provide high signal-to-noise in measurements of physiology and gene expression (31).

Recently, we documented the physiology and gene expression profile of synchronized Chlamydomonas populations acclimated to diurnal cycles of LL, moderate light (ML), and HL (50, 200, and 1,000 µmol photons m^–2^ s^–1^, respectively) (30). We showed that photoacclimatory phenotypes persist in the night phase. Even after 10 h of darkness, populations acclimated to diurnal LL exhibited high Chl content, LHC protein abundance, and tightly stacked thylakoid membranes, while HL-acclimated populations maintained relatively loose stacks of thylakoid membranes, low photosystem and antenna protein abundance, and high NPQ capacity. We hypothesized that maintenance of these physiological states may be beneficial for cells that routinely encounter dim days or bright days, but that it likely decreases fitness upon a change in daylight intensity. To test this hypothesis, we transitioned LL-acclimated populations to diurnal HL and HL-acclimated populations to diurnal LL. We show that Chlamydomonas is flexible and can reacclimate in just 1 d despite severe photoinhibition. Using transcriptomics and proteomics, we document the gene expression program for reacclimation with high temporal resolution over two diurnal cycles.

## Results

### LL-Acclimated Cells Achieve Impressive Growth and Recovery during Their First Day in HL Despite Severe Photoinhibition, while Acclimation to HL Stunts Growth upon a Transition to Diurnal LL.

**Fig. 1.**
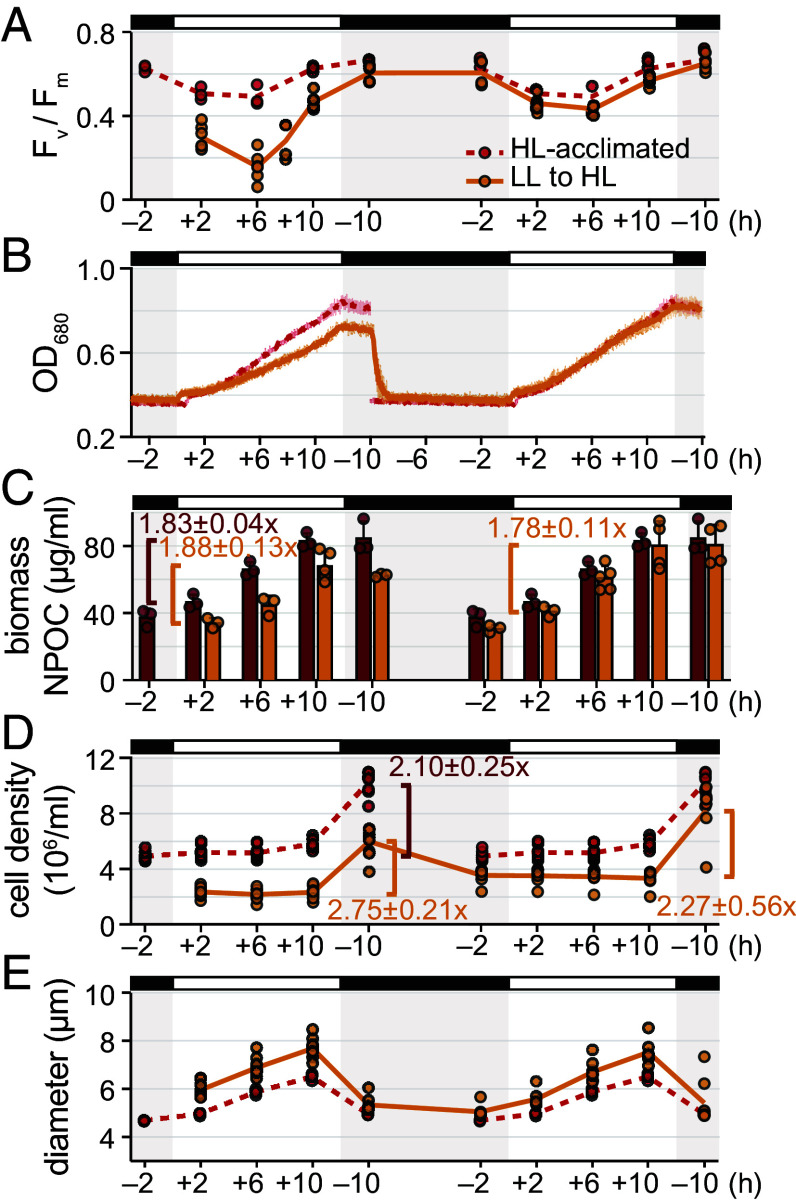
LL-acclimated cells achieve impressive growth and recovery during their first day in HL despite severe photoinhibition. The physiology of LL-acclimated cells transitioned to diurnal HL (orange) was monitored for two diurnal cycles. Results for HL-acclimated populations (red) are reproduced from Dupuis et al. (30). Lines and bars represent the mean of experimental replicates. (*A*) Maximum photochemical efficiency of PSII (F_v_/F_m_). (*B*) OD_680_ monitored continuously. Error bars represent the SD from the mean. (*C*) NPOC concentration of biomass in the cultures. Error bars represent the SD from the mean. Fold-changes during the light phase are noted above the brackets as mean ± SD. (*D*) Cell density. Fold-changes over the day are noted near the brackets as mean ± SD. (*E*) Mean cell diameter across the populations.

To determine the kinetics of diurnal photoacclimation in Chlamydomonas, we acclimated populations to diurnal LL or HL using the conditions established in the previous study (30), and then surprised them with the opposite light (LL to HL, and HL to LL). We monitored the populations for two consecutive diurnal cycles, diluting the populations to the starting density after the first day (*SI Appendix*, Fig. S1). To test whether acclimation to diurnal LL decreased fitness in diurnal HL, we monitored photosynthetic efficiency and growth. The maximum efficiency of PSII (F_v_/F_m_) of the transitioned cells plummeted from 0.67 ± 0.02 prior to the transition to 0.16 ± 0.07 in the middle of the first HL day, indicating severe photoinhibition (Fig. 1*A*) (30). Then, we observed an impressive recovery in the latter half of the day. By the night phase, the F_v_/F_m_ had rebounded to 0.61 ± 0.04 and it then tracked closely with that of populations that had been acclimated to diurnal HL for several weeks.

We found that optical density (OD_680_) increased less for LL-acclimated populations during a surprise HL day than for populations acclimated to diurnal HL (Fig. 1*B*). Yet, by just their second day in HL, the previously LL-acclimated cultures achieved the same increase in OD_680_ as did HL-acclimated cultures.

Since Chl content may influence OD_680_, we measured nonpurgeable organic carbon (NPOC) content as another proxy for biomass. Although all cultures were adjusted to an OD_680_ near 0.4 at the beginning of the experiment, the starting NPOC biomass content and number of cells in the transitioned cultures were lower than in the acclimated cultures (Fig. 1 *C* and *D*). However, the fold-change in the NPOC biomass content of transitioned cultures over the first HL day was similar to that of HL-acclimated cultures. Thus, LL-acclimated cells could achieve similar increases in biomass over a surprise HL day despite the severe photoinhibition.

LL-acclimated cells were larger than the HL-acclimated cells across the 2-d time course, particularly prior to the first division event (Fig. 1*E*). The LL-to-HL transitioned cells successfully divided at the end of their first day in HL, and in fact, achieved a greater fold-change in cell density than did the HL-acclimated cells (Fig. 1*D*). These results suggest that although acclimation to LL causes severe photoinhibition during a surprise HL day, it results in greater vegetative growth of Chlamydomonas than is achieved by cells that are long-term acclimated to diurnal HL.

We also determined how acclimation to diurnal HL influenced Chlamydomonas’ fitness upon a transition to diurnal LL. HL-acclimated populations exhibited high F_v_/F_m_ in LL and achieved similar increases in OD_680_ and NPOC content as did LL-acclimated populations (*SI Appendix*, Fig. S2 *A–C*). However, the HL-to-LL transitioned cells were unable to sufficiently increase their size and hence failed to divide after their first day in LL (*SI Appendix*, Fig. S2 *D* and *E*). Thus, acclimation to diurnal HL limits growth during LL days, decreasing fitness upon a change in the diurnal light environment. By the second day, the HL-to-LL cells became larger like LL-acclimated cells did, and some cells successfully divided at the light-to-dark transition.

### LL-Acclimated Cells Alter Their Thylakoid Membrane Architecture and Exhibit Signs of Chloroplast Swelling upon a Transition to HL.

Components of the PSII repair pathway (e.g., FTSH protease, STL1 kinase, chloroplast ribosomes) are sterically excluded from appressed membrane regions where PSII is localized (17, 39, 40). Decreased stacking of appressed thylakoid membranes is thought to be important for PSII repair in prolonged HL (17, 41). Stacking is driven by electrostatic forces and van der Waals interactions between the lipids and proteins of adjacent membranes (42). Thus, changes in LHCII abundance, their phosphorylation by STT7 kinase, and the osmolarity of the chloroplast stroma and thylakoid lumen can alter thylakoid stacking (35, 43–45). Therefore, we tested whether the recovery in F_v_/F_m_ observed in the LL-to-HL transition might be accompanied by changes in thylakoid membrane stacking.

**Fig. 2.**
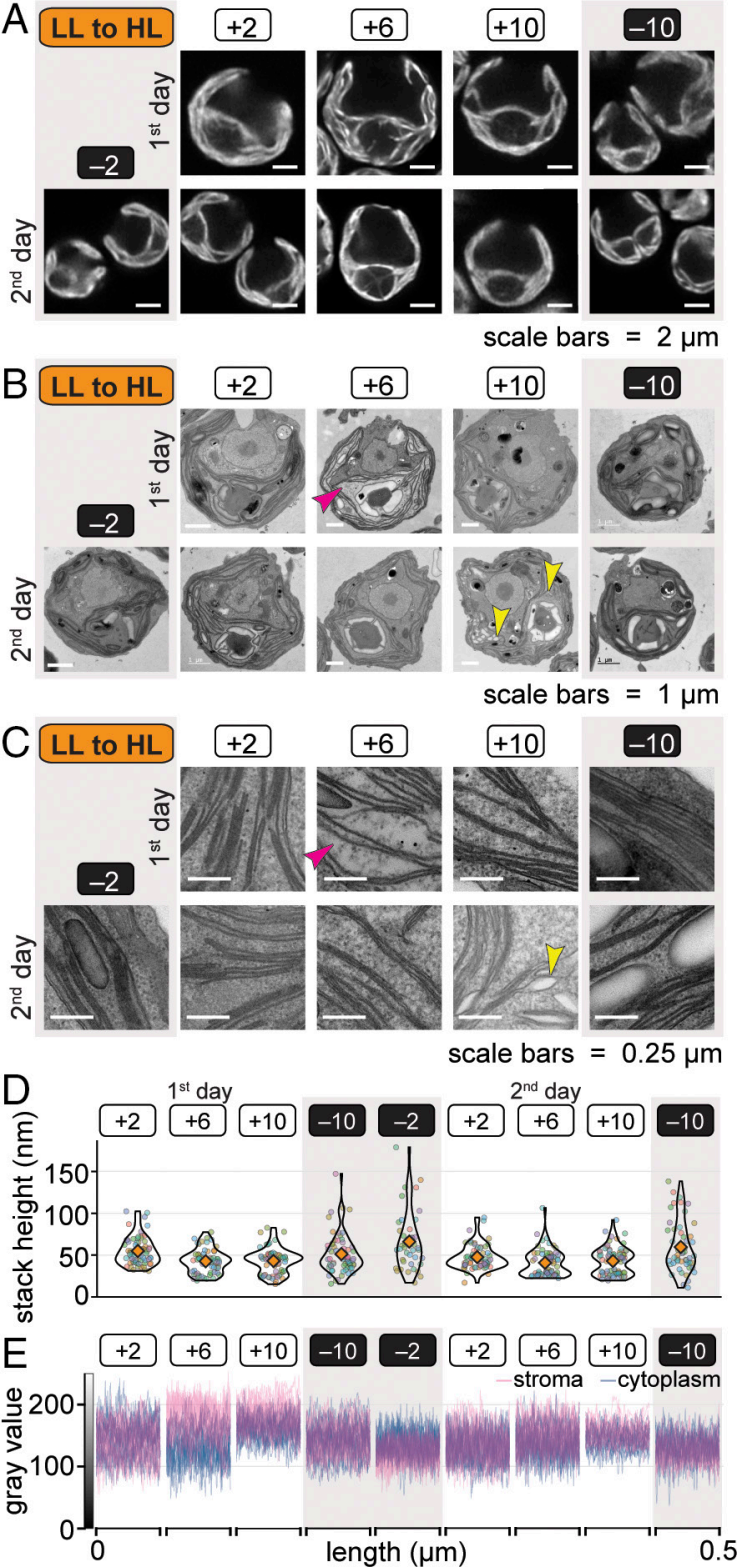
LL-acclimated cells rapidly alter their thylakoid membrane architecture and exhibit signs of chloroplast swelling upon a transition to diurnal HL. (*A*) Chl autofluorescence of representative live cells during the transition from diurnal LL to HL imaged by confocal Airyscan microscopy. Each Chlamydomonas cell has a single cup-shaped chloroplast with a pyrenoid at its base. (*B*) Representative electron micrographs of fixed cells during the transition from diurnal LL to HL. The pink arrowhead indicates the “light stroma” phenotype. Yellow arrowheads indicate examples of swollen thylakoids. (*C*) Representative electron micrographs of thylakoid membranes, with notable phenotypes indicated as in (*B*). (*D*) Height of thylakoid membrane stacks in electron micrographs of at least ten representative cells per timepoint during the transition from diurnal LL to HL. Measurements of individual thylakoid membrane stacks are shown as circles colored by cell. Orange diamonds represent the mean. (*E*) Gray value profiles (0 to 255) across the length of two 0.5 µm lines through the chloroplast stroma (pink) and cytoplasm (blue) in electron micrographs of ten representative cells per timepoint during the transition from diurnal LL to HL. When the profiles of the stroma overlap with those of the cytoplasm, the color appears purple.

We obtained subdiffraction-resolution micrographs of Chl autofluorescence in live Chlamydomonas cells during the transition using confocal Airyscan microscopy. At the beginning of the HL day, the chloroplasts of LL-acclimated cells contained thick regions of Chl throughout the lobes and base, as has been reported previously for LL-acclimated Chlamydomonas (Fig. 2*A* and *SI Appendix*, Fig. S3) (30, 35). Then, by the first +6 timepoint, the chloroplast displayed discrete, thin structures of Chl fluorescence, suggesting a decrease in thylakoid membrane stacking. During the second day in HL, the fluorescent structures appeared less dispersed than they had in the first HL afternoon but still appeared thinner than at the start of the transition.

As a complementary approach, we fixed cells at each timepoint during the LL-to-HL transition and imaged them by transmission electron microscopy (TEM) (Fig. 2 *B* and *C*). Changes in the height of thylakoid membrane stacks and their gray value profiles in the electron micrographs confirmed that stacking decreased substantially between the +2 and +6 timepoints of the first HL day (Fig. 2*D* and *SI Appendix*, Fig. S4).

Across the population, the stroma of the chloroplast appeared less dense with ribosomes and other large densities at the first +6 timepoint when we observed the lowest F_v_/F_m_ (Fig. 2 *B* and *C*, pink arrowheads, and Fig. 1*A*). This was also evident as a striking distinction between the gray value profiles of the chloroplast stroma and the cytoplasm of the same cells, which was rarely observed at any other time (Fig. 2*E* and *SI Appendix*, Fig. S5). This light stroma phenotype suggests that the entire chloroplast is swollen at the first +6 timepoint of the transition. Chloroplast swelling has been documented in *Arabidopsis* during HL stress as a result of damage to the chloroplast envelope and changes in osmolarity (46, 47). Changes in the osmolarity of the stroma could impact thylakoid membrane appression (48, 49).

Besides changes in stacking and the appearance of the stroma, we also observed swollen thylakoids in some cells of the LL-to-HL population, particularly at the +10 timepoints (Fig. 2 *B* and *C*, yellow arrowheads). Thylakoid swelling has been reported in Chlamydomonas exposed to photoinhibitory light intensities and has been attributed to increased accumulation of ammonium ions in the thylakoid lumen upon acidification and cyclic electron flow (50–52).

### Global View of Gene Expression Reveals Widespread mRNA Induction at the Onset of a Surprising HL Day.

**Fig. 3.**
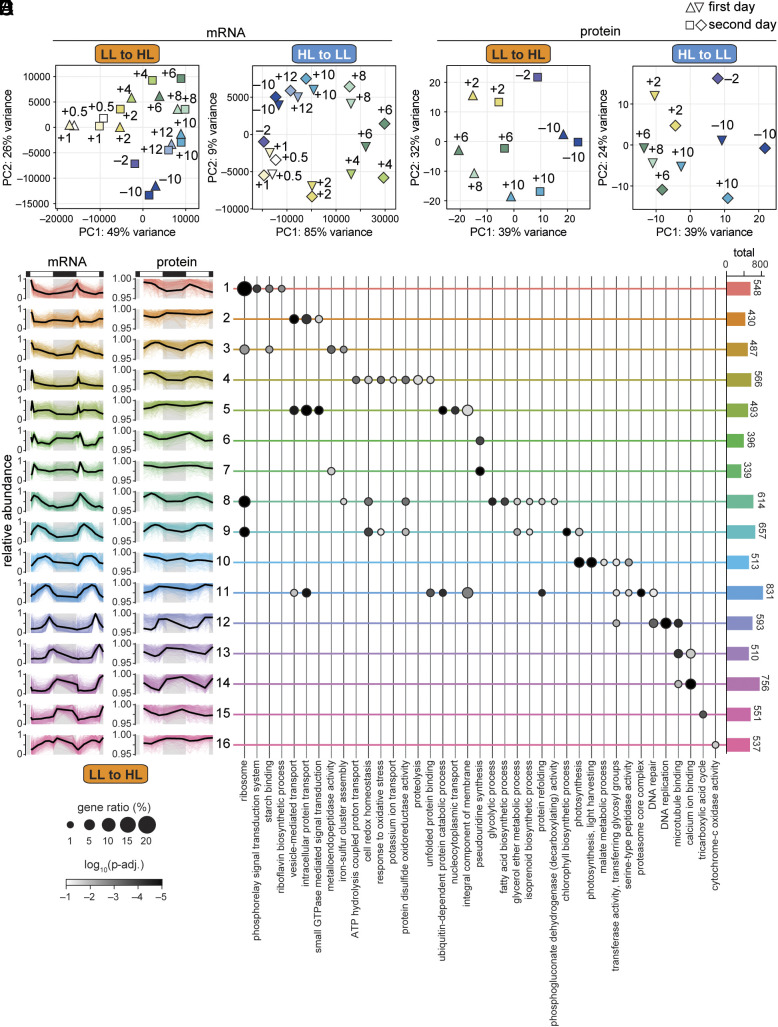
Global view of gene expression reveals maintenance of diurnal program, as well as widespread mRNA induction at the onset of a surprising HL day. (*A*) PCA of the transcriptome of cells transitioned from diurnal LL to HL at 19 timepoints. Samples collected on the first day are indicated as triangles, and those collected on the second day as squares. Points are colored and labeled according to the time of day. (*B*) PCA of the transcriptome of cells transitioned from diurnal HL to LL at 19 timepoints. Samples collected on the first day are indicated as triangles, and those collected on the second day as diamonds. Points are colored and labeled according to the time of day as in (*A*). (*C*) PCA of the proteome of cells transitioned from diurnal LL to HL at 10 timepoints, represented as in (*A*). (*D*) PCA of the proteome of cells transitioned from diurnal HL to LL as in (*B*). (*E*) Pattern of mRNA (*Left*) and protein (*Right*) accumulation for 16 distinct clusters of coexpressing genes during the LL-to-HL transition. Abundance was normalized relative to the maximum for each gene during the LL-to-HL transition. Colored lines show the relative abundance of individual mRNAs and proteins, while the black line shows the mean relative abundance. Cluster assignment is available in Dataset S2. (*F*) Enrichment of representative GO terms in the 16 clusters of genes. Dot size indicates the fraction of genes in each cluster with this GO assignment, dot shading indicates the log_10_(*P*-adj.) of enrichment, and bars at the *Right* show the total number of genes in each cluster. The full list of significantly enriched GO terms is available in Dataset S3.

To understand the molecular events underlying the recovery in photosynthesis and chloroplast integrity, and to further explore photoacclimation processes in Chlamydomonas, we monitored the transcriptome and proteome of the LL- and HL-acclimated populations as they transitioned to a new intensity of diurnal light (Datasets S1 and S2). We began collecting RNA samples 30 min after lights-on (+0.5), as we anticipated a rapid transcriptional response to a surprising quantity of light. Principal component analysis (PCA) of the transcriptome over the 2-d LL-to-HL transition separated the data primarily by time of day, as observed previously for LL- and HL-acclimated Chlamydomonas (Fig. 3*A*) (30). Samples collected at the same times from the first day (triangles) and second day (squares) in HL were mostly positioned near each other. However, the transcriptome at the first +0.5 and +1 timepoints was quite distinct from the same times on the second HL day, as well as from the subsequent timepoint (first +2) on PC1, which represented half of the variation in the data. No such distinction between these timepoints was apparent in PC1 of the HL-to-LL transition, which represented 85% of the variation in the transcriptome (Fig. 3*B*).

Time of day also explained the majority of the variation in the proteome for both transitions (Fig. 3 *C* and *D*), as observed previously (30). Of the timepoints when the proteome was examined, the +6 timepoint appeared the most distinct between the first and second day in HL.

We investigated the changes in mRNA abundances that occur in LL-acclimated cells at the onset of the first HL day and how these changes shape the proteome. We detected 8,821 cognate mRNAs and proteins across the full time course and grouped these into 16 clusters based on how their relative abundances changed over time (Fig. 3*E* and Dataset S2). mRNAs in many of the clusters exhibited a sharp peak in abundance within the first 2 h of HL (Clusters 4 to 7, ~20% of the mRNAs included in the analysis). mRNAs in these clusters often exhibited a similar abundance peak during the second HL day, though its magnitude often differed from that of the first HL day. This induction was not evident in any groups of coexpressing mRNAs during the onset of the HL-to-LL transition (*SI Appendix*, Fig. S6 and Dataset S2). However, many mRNAs did exhibit such an induction at lights-on during the second day of the HL-to-LL transition.

Among the mRNAs that peak within the first 2 h of the LL-to-HL transition were genes enriched for Gene Ontology (GO) terms related to oxidative stress, ion transport, and proteolysis (Cluster 4 genes, whose proteins were most abundant midday) (Fig. 3 *E* and *F* and Dataset S3). Genes in Cluster 5, whose proteins accumulated in the latter half of the day, were enriched for vesicular transport, protein transport, and ubiquitin related GO terms. Clusters 6 and 7 were enriched for terms related to metalloendopeptidases, transcription, and RNA modification. Thus, the mRNA induction at the start of the transition supports redox homeostasis, proteostasis, and housekeeping functions.

This global view of the gene expression landscape also revealed adjustments to the proteome in the second day of the LL-to-HL transition relative to the first. Proteins in Clusters 1, 4, 8, and 10 often appeared less abundant the second day, and were enriched for photosynthesis, light harvesting, and starch metabolism GO terms (Fig. 3 *E* and *F*, *SI Appendix*, Fig. S7, and Dataset S3). Proteins in Clusters 5 and 11 often appeared more abundant in the second day in HL, and were enriched for functions in proteostasis.

Taken together, we find that Chlamydomonas’ rhythmic gene expression program is maintained in severe light stress and that the surprise of excess light can induce widespread transcriptional changes that may be important for restoring homeostasis after photodamage without disrupting the diurnal program.

### NPQ Mechanisms Are Activated within the First HL Day.

**Fig. 4.**
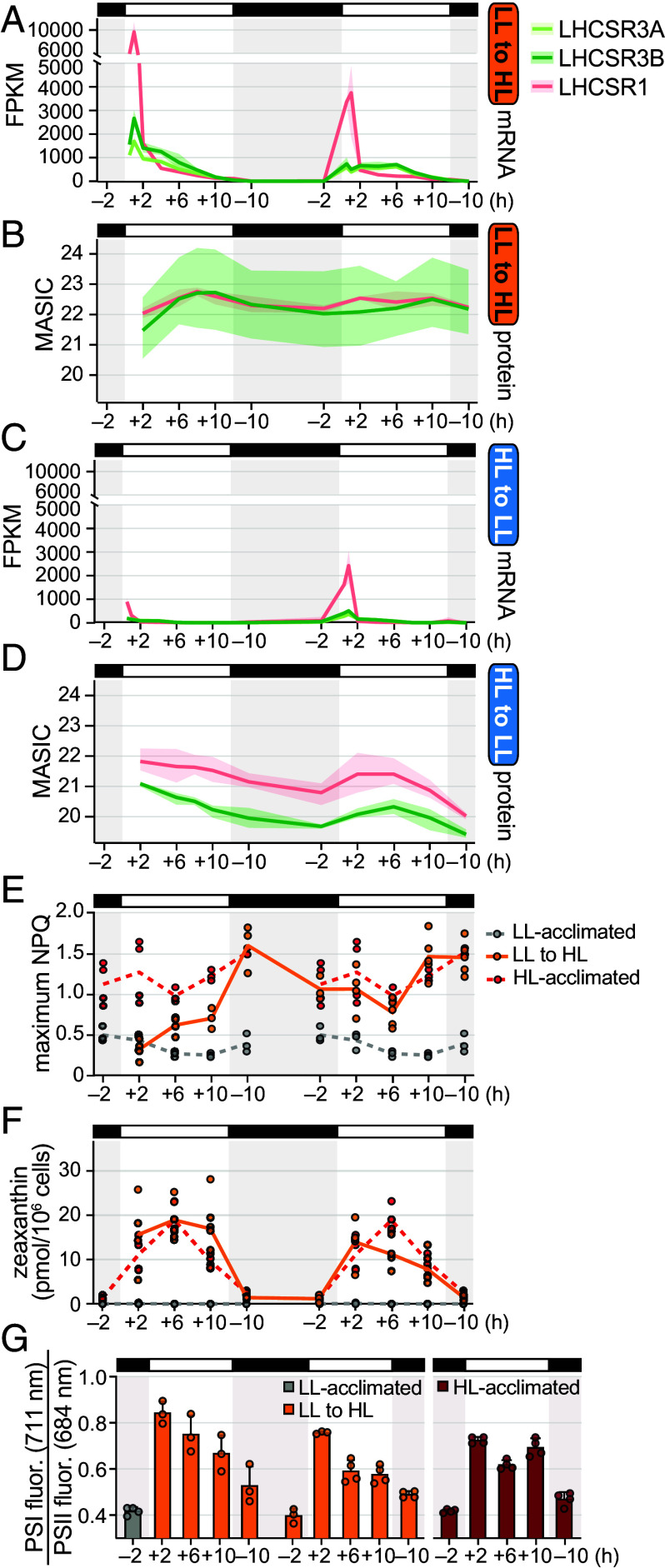
NPQ mechanisms are activated within the first HL day. Gene expression data are represented as the mean expression across the experimental replicates and the 95% CI. Results for HL-acclimated populations (red) and LL-acclimated populations (gray) are reproduced from ref. 30. (*A*) Abundance (FPKM) of LHCSR transcripts during the diurnal LL-to-HL transition. (*B*) Abundance (MASIC values) of LHCSR proteins during the diurnal LL-to-HL transition. (*C*) Abundance (FPKM) of LHCSR transcripts during the diurnal HL-to-LL transition. (*D*) Abundance (MASIC values) of LHCSR proteins during the diurnal HL-to-LL transition. (*E*) Maximum NPQ measured over 50 to 1,500 µmol photons m^–2^ s^–1^ at each timepoint. Lines represent the mean of the experimental replicates. (*F*) Cellular zeaxanthin content. Lines represent the mean of the experimental replicates. (*G*) Relative Chl fluorescence from PSI (at 711 nm) to PSII (at 684 nm) measured at 77 K. Error bars represent the SD from the mean.

Unsurprisingly, genes encoding photoprotective functions were among the genes that exhibited mRNA induction at the onset of the transition from diurnal LL to HL. The expression of qE-related genes *LHCSRs* and *PSBSs* was previously reported to peak at lights-on in diurnal ML and LL (200 and 60 µmol photons m^–2^ s^–1^, respectively), with the magnitude of expression being correlated to the intensity of light (31). We found that the induction of the *LHCSRs* was much higher during lights-on at the first HL period than at the second (Fig. 4*A*). Large increases in mRNA abundance at the first instance of HL may enable de novo accumulation of LHCSR3 and LHCSR1 proteins in the LL-acclimated cells, which steadily accumulated over the first HL day (Fig. 4*B*). Conversely, HL-acclimated cells transitioned to LL did not induce *LHCSRs* during the first LL morning, but did during the second (Fig. 4*C*), and LHCSR protein abundance decreased over the first diurnal cycle of LL (Fig. 4*D*).

To determine how these gene expression changes influenced photoprotective quenching, we first monitored NPQ capacity during the transitions. In both cases, NPQ capacity was correlated with LHCSR accumulation. LL-acclimated cells increased their NPQ capacity from 0.31 ± 0.12 at the first +2 timepoint in HL to 0.70 ± 0.13 at the first +10, and by the dark phase, had reached the capacity of cells that had been acclimated to HL for several weeks (1.60 ± 0.30) (Fig. 4*E*). Conversely, the NPQ capacity of HL-acclimated cells transitioned to LL decreased as LHCSR abundance decreased over the first day (*SI Appendix*, Fig. S8*A*).

Upon HL exposure, a high ∆pH across the thylakoid membrane activates CVDE1, which converts violaxanthin to the quenching pigment zeaxanthin. HPLC measurements showed that LL-acclimated cells were competent to accumulate zeaxanthin to the same degree as HL-acclimated cells do by the first +2 in HL (Fig. 4*F*). Their de-epoxidation state followed a similar pattern as in the HL-acclimated cells (*SI Appendix*, Fig. S8*C*). HL-acclimated cells were also competent to adjust zeaxanthin levels to LL-acclimated levels by the first +2 of a surprising LL day (*SI Appendix*, Fig. S8 *B* and *C*).

We also investigated state transitions in the populations by measuring Chl fluorescence spectra at 77 K. Within minutes of HL, STT7 kinase is known to phosphorylate LHCII trimers, increasing their association with PSI and thereby redistributing excitation energy between PSI and PSII (53, 54). We found that while LL-acclimated cells maintained the relative fluorescence of PSI:PSII near 0.4 at the beginning of the LL day (30), cells transitioned to HL doubled their relative PSI:PSII fluorescence to 0.89 ± 0.05 within the first 2 h (Fig. 4*G*). This increase was reversible, decreasing again in the night phase as it does for HL-acclimated cells. Such increases in PSI:PSII fluorescence may reflect not only association of LHCII with PSI, but also PSII damage.

In summary, these data suggest that LL-acclimated cells are competent to perform zeaxanthin-dependent quenching (qZ) and state transition-dependent quenching (qT) within just 2 h of a HL day. After several more hours, they accumulate LHCSR protein and increase their overall NPQ capacity, which may be aided by superinduction of *LHCSR* mRNAs at lights-on of the transition. This gradual increase in overall NPQ capacity may contribute to the observed recovery in F_v_/F_m_ (Fig. 1*A*).

### Transient Depletion and Delayed Accumulation of Photosystem Complex Transcripts May Mediate the Reduction in Light Harvesting During Acclimation to Diurnal HL.

**Fig. 5.**
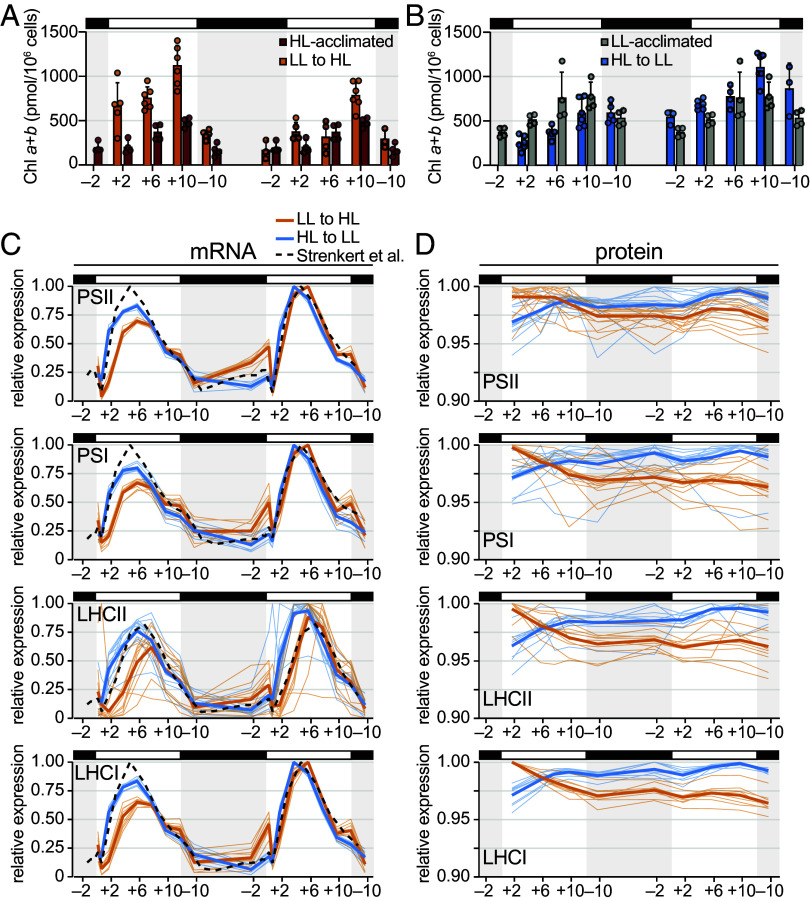
Transient depletion and delayed accumulation of photosystem complex transcripts may reduce absorptive capacity during acclimation to diurnal HL. Results for HL-acclimated populations (red) and LL-acclimated populations (gray) are reproduced from Dupuis et al. (30). Error bars represent the SD from the mean. mRNA and protein abundances were normalized relative to the maximum abundance observed in each population (abundance in LL-to-HL normalized relative to the maximum abundance observed in LL-to-HL; abundance in HL-to-LL normalized relative to the maximum abundance observed in HL-to-LL). Fine lines show the relative abundance of individual mRNAs and proteins as the mean across the experimental replicates, and the thick lines show the mean across all detected mRNAs or proteins of the photosystems and their antenna. (*A*) Cellular Chl content during the transition from diurnal LL to HL. (*B*) Cellular Chl content during the transition from diurnal HL to LL. (*C*) Changes in photosystem and antenna mRNA abundances during the diurnal transitions compared to that of ML-acclimated cells reported by Strenkert et al. (31) (dashed lines). (*D*) Changes in photosystem and antenna protein abundances during the diurnal transitions.

Next, we sought to determine how Chlamydomonas’ light-harvesting capacity and photosystem abundance are adjusted during the diurnal transitions. HPLC measurements showed that LL-acclimated cells transitioned to HL had higher Chl content than HL-acclimated cells during the first HL day (Fig. 5*A*), explaining why adjusting the cultures to the same OD_680_ caused the LL-to-HL cultures to start with lower biomass (Fig. 1). After the cells divided (Fig. 1), their Chl content became more similar to that of HL-acclimated cells, and throughout the second HL day, it was lower than it had been the previous day. Conversely, HL-acclimated cells had lower Chl content than LL-acclimated cells had during their first day in LL (Fig. 5*B*). As the HL-to-LL transitioned cells failed to divide after the first LL day and remained relatively large during the dark phase (*SI Appendix*, Fig. S2), they began the second LL day with a higher cellular Chl content than the LL-acclimated cells did. They continued to increase their Chl content over the second day of the transition.

We found that transcripts for both photosystems and their respective LHCs sharply decreased in abundance during the first hours of the LL-to-HL transition (Fig. 5*C*). A slight trough in PSII and LHCII mRNA abundance can also be observed in cells acclimated to ML (31) and in the HL-acclimated cells on the second LL day. In addition, mRNA accumulation was delayed during the first day of the LL-to-HL transition relative to diurnal ML conditions, whereas they accumulated on time or even earlier during the HL-to-LL transition.

The abundance of the cognate PSI, LHCII, and LHCI proteins showed a concerted decrease over the first day of the LL-to-HL transition (Fig. 5*D*), as did the PSII subunits of the oxygen-evolving complex (*SI Appendix*, Fig. S9*A*). This could reflect protein degradation and/or dilution if there is not commensurate synthesis as the cell volume increases over the light phase. The PSII core subunits PsbA (D1), PsbD (D2), PsbC (CP43), and PsbB (CP47) did not begin to decrease until later in the first HL day (*SI Appendix*, Fig. S9*A*), consistent with reports that translation of these subunits transiently increases upon HL exposure to support PSII repair (11, 55–57). Conversely, the abundances of the photosystem and antenna proteins increased over the HL-to-LL transition (Fig. 5*D* and *SI Appendix*, Fig. S9*B*). Collectively, these results suggest that changes in photosystem and antenna abundance are likely driven by changes not only in synthesis but also in degradation.

### Populations Acclimated to Diurnal LL Promptly Induce Chaperones, Proteases, and the Autophagy Pathway during a Surprise HL Morning.

**Fig. 6.**
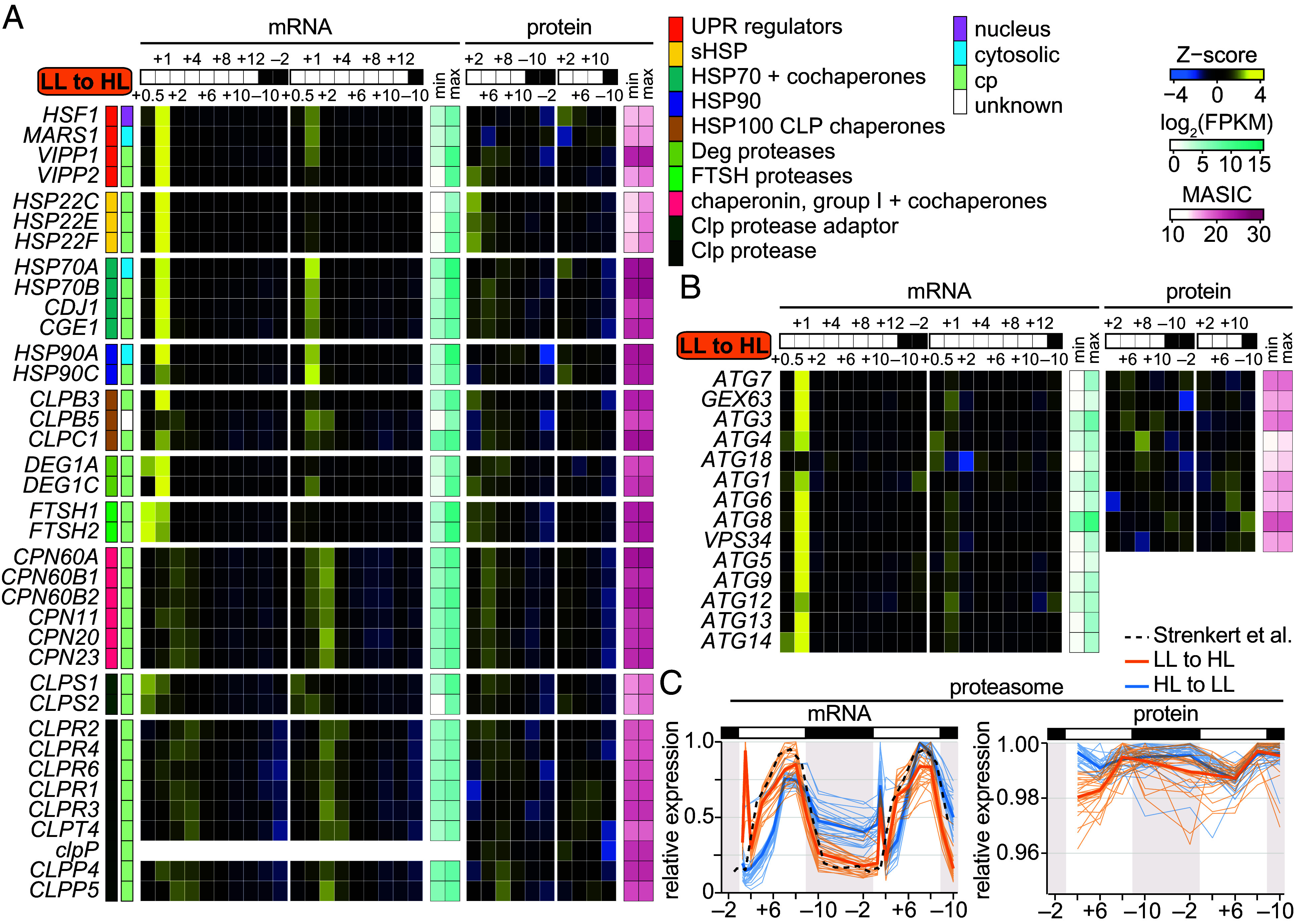
Chaperones, proteases, and the autophagy pathway are rapidly induced in LL-acclimated cells transitioned to diurnal HL. (*A*) Changes in mRNAs and proteins involved in chloroplast protein homeostasis. Z-scores of mean mRNA abundances (FPKM) and Z-scores of mean protein abundances (MASIC values) are used to show patterns over time. Minimum and maximum FPKM and MASIC values are also shown to demonstrate the dynamic range. Protein localization is listed according to the review by Schroda and deVitry 2023. (*B*) Changes in autophagy-related mRNAs and proteins represented as in (*A*). (*C*) Expression of proteasome genes during the diurnal transitions compared to that of ML-acclimated cells reported by Strenkert et al. (31), represented as in Fig. 5. Fine lines show the relative abundances of individual mRNAs and proteins as the mean across the experimental replicates, and the thick lines show the mean across all detected mRNAs or proteins.

To distinguish degradation events during the transition from diurnal LL to HL, we investigated the expression of proteases, chaperones, and their regulators. LL-acclimated cells had the transcriptional signature of a heat shock or unfolded protein response during the first hour of HL (Fig. 6*A*). Target loci of the heat shock transcription factor HSF1, including the *HSF1* locus itself, loci of the cytosolic chaperones *HSP90A* and *HSP70A*, and loci of the chloroplast chaperones *HSP22C*, *HSP22E*, *HSP22F*, and *HSP70B* exhibited high mRNA abundances at the +1 timepoint, particularly during the first day of the transition (58, 59). The same was true for several cochaperones and HSP100s; the *MARS1* kinase, which has been proposed to regulate the chloroplast unfolded protein response through retrograde signaling (60); and the VIPP paralogs, which are thought to play a role in the response to chloroplast membrane stress (52, 61). In addition, transcripts encoding the soluble chloroplast serine endopeptidase DEG1A; DEG1C; and the FTSH1 and FTSH2 subunits of the thylakoid-membrane-tethered processive metalloprotease, which degrade the D1 polypeptide during PSII repair (62, 63), were also increased within the first hour of the transition. Transcriptional induction of several of these genes has been reported previously upon a dark-to-light transition or under continuous HL (60, 61, 63–65). However, the level of induction measured in the LL-to-HL population was not observed in the HL-to-LL transition (*SI Appendix*, Fig. S10) nor in the ML-acclimated populations studied previously (31).

Proteomics showed that the VIPP proteins, chaperones, and proteases were most abundant between the +2 and +8 timepoints of the first day of HL (Fig. 6*A*), concomitant with the striking changes in chloroplast ultrastructure (Fig. 2). The chloroplast Group I chaperonins, their cochaperones, the Clp protease adaptor CLPS1, and the Clp monomers CLPR2 and CLPR4 also peaked in abundance during this period, although transcripts for these proteins accumulated more gradually over the first day of the transition rather than as a sharp induction at lights-on.

Under photooxidative and proteotoxic stress in the chloroplast, Chlamydomonas has also been reported to induce autophagy-related genes (65–67). We observed that several autophagy genes were induced at lights-on of the LL-to-HL transition (Fig. 6*B*). We also observed a sharp induction of proteasome mRNAs at the beginning of the LL-to-HL transition, which was roughly 2× higher than that observed during the HL-to-LL transition and was absent in ML-acclimated cells (Fig. 6*C*) (31). Although some of these mRNAs exhibited peaks in abundance in the second morning of the HL-to-LL transition and in ML-acclimated cells (31), the peaks were of substantially lower magnitude. For example, transcripts encoding ATG8, a protein critical for autophagosome formation that is often used as a marker for autophagy (68, 69), increased to ~1,700 FPKM at the first +1 timepoint of the LL-to-HL transition but only ~700 FPKM in the HL-to-LL transition.

The proteins involved in autophagy were not coexpressed, but given that many of them are degraded along with the autophagic cargo (70), this was not unexpected. The abundance of the proteasome subunits on the other hand increased gradually over the first HL day. Proteasome function feeds back on de novo protein synthesis and is necessary for efficient recovery from photoinhibition in Chlamydomonas (71). It has also been linked to chloroplast proteostasis (72). Taken together, rapid induction of chaperones, proteases, and the autophagy pathway could be important for repair and remodeling during the transition from diurnal LL to HL.

## Discussion

We have undertaken a systems analysis of synchronized Chlamydomonas populations acclimated to dim days and bright days as they adjust to an unanticipated diurnal light intensity. Chlamydomonas populations acclimated to diurnal LL exhibit robust growth in a surprise HL day, achieving similar biomass yields over their first day in HL as do populations acclimated to diurnal HL through weeks of exposure (Fig. 1). This is despite severe photoinhibition and apparent chloroplast swelling that occur during the first 6 h of a surprise HL day (Figs. 1 and 2). LL-acclimated cells can recover PSII efficiency by the end of their first HL day, and by their second day, they perform almost indistinguishably from cells that are long-term accustomed to this daylight intensity. In contrast, cells acclimated to diurnal HL suffer growth limitation and do not divide after a surprise LL day (*SI Appendix*, Fig. S2), but these cells also adjust their physiology to improve growth yields just 1 d later. Taken together, these results demonstrate that Chlamydomonas is resilient to challenging light intensities under diurnal cycles and is competent to acclimate to variation in daylight intensities within 48 h.

Using the synchronous cell system, we have documented temporally resolved changes in PSII photochemistry (F_v_/F_m_), thylakoid membrane stacking, PSII subunit abundance, and the abundance of chloroplast proteases during the transition from LL to HL. These data are compatible with the sequence of events in which light-induced damage to PSII precedes a decrease in thylakoid membrane stacking and initiation of the PSII repair cycle, including degradation and de novo synthesis of PSII core subunits (41, 73).

Microscopy of live and fixed Chlamydomonas cells showed that LL-acclimated cells decreased thylakoid membrane stacking within the first 6 h of HL (Fig. 2 and *SI Appendix*, Figs. S3 and S4). Decreased stacking may allow the PSII repair machinery to access damaged PSII in these cells, providing a plausible mechanism for the recovery in F_v_/F_m_ that we observed in the latter half of the day (17, 41). On the other hand, segregation of the two photosystems is thought to prevent excitation energy spillover (74). Thus, it is also possible that rapid unstacking may result in spillover midday and contribute to the decrease in PSII efficiency.

Unlike at other timepoints, the chloroplast stroma appeared less densely packed with ribosomes than the cytoplasm did at the first +6 timepoint in HL, suggesting that the chloroplasts were swollen (Fig. 2 and *SI Appendix*, Fig. S5). As thylakoid membrane stacking is driven by electrostatic forces, changes in the osmolarity of the stroma could be related to the changes in thylakoid membrane appression that we observed during the first HL day (48, 49). Chloroplast swelling has been documented in *Arabidopsis* cells during photoinhibition and apparent damage to the chloroplast envelope (46), and the swollen chloroplasts are cleared through microautophagy of the entire organelle (chlorophagy) (46, 47, 75). We found that the light stroma phenotype was largely resolved across the Chlamydomonas LL-to-HL population within just 4 h. Chlamydomonas does not possess a canonical microautophagy pathway, nor is it likely that the cell’s only chloroplast would be completely turned over in just 4 h. However, piecemeal macroautophagy could play a role in the recovery of chloroplast integrity. Autophagy genes and others enriched for the “vesicle-mediated transport” GO term were induced at the onset of the LL-to-HL transition (Figs. 3 and 6).

The LL-acclimated cells exhibited the transcriptional signature of the chloroplast unfolded protein response at the start of the transition (Fig. 6). Several chaperones, proteases, and the VIPP proteins accumulated midday. Overexpression of VIPP1 suppresses chloroplast swelling in *Arabidopsis* (46). The VIPP proteins interact with the chloroplast chaperone HSP70B in Chlamydomonas and have been proposed to sense damaged or misfolded proteins in the thylakoid membranes, recruit chaperones and proteases to such sites, and contribute to membrane remodeling (52, 61, 76, 77). Future work should 1) confirm whether the light stroma phenotype in fact reflects chloroplast swelling, and 2) determine the contributions of autophagy, proteostasis pathways, and VIPP proteins in the rapid restoration of chloroplast form and function during a surprise HL day. For example, activation of autophagy could be assessed by monitoring ATG8 lipidation (69), and the capacity to acclimate to diurnal HL without induction of the chloroplast unfolded protein response (e.g., in *HSF1*-RNAi lines and *mars1* mutant lines) could be tested (58–60).

Among the proteases induced at the beginning of the diurnal LL-to-HL transition were FTSH1, FTSH2, and DEG1 (Fig. 6). These proteases cooperatively degrade the D1 polypeptide (62, 63, 78). However, the PSII core subunits PsbA (D1), PsbD (D2), PsbC (CP43), and PsbB (CP47) increased during the first HL morning, while the abundance of many other photosystem subunits and LHC proteins decreased (Fig. 5 and *SI Appendix*, Fig. S9), suggesting net synthesis as has been documented previously (11, 55–57). Previous reports have shown that *psbA* transcription increases upon a transition from dark to light, as does the degradation rate of *psbA* mRNAs and several other chloroplast-encoded mRNAs (79). RNA sequencing (RNA-Seq) libraries prepared using poly-A selection do not quantitatively recover chloroplast-encoded mRNAs. However, the nucleus-encoded PSII mRNAs did seem to decrease at lights-on, particularly in cells transitioned from LL to HL (Fig. 5) (31). mRNAs encoding LHCII, PSI, and LHCI proteins also appeared to be depleted at the onset of the first HL day, and their accumulation over the light phase was delayed relative to populations maintained in diurnal ML (Fig. 5). HL-dependent decreases in transcription, translation, and half-life of *LHCB* mRNAs have been demonstrated in Chlamydomonas (13, 44, 80, 81). We hypothesize that the apparent depletion of nucleus-encoded *PSB*, *LHCBM*, *PSA*, and *LHCA* mRNAs reflects other such light-responsive decreases in mRNA half-life.

Dark-to-light transitions are known to induce *LHCSR* and *PSBS* expression in Chlamydomonas, and the magnitude of mRNA accumulation at this time depends on the light intensity (3, 31). Here, we have found that the level of induction also depends on the light intensity experienced the previous day (Fig. 4). LL-to-HL cells had much higher levels of these mRNAs at the first dark-to-light transition, whereas HL-to-LL cells increased their induction on the second dark-to-light transition. This dependence on the prior day’s light environment could stem, in part, from differences in the light absorption capacity and resulting photosynthetic electron transfer. Inhibition of photosynthetic electron transfer by DCMU has been shown to suppress the induction of *LHCSR3A* (3, 26). Thus, HL-acclimated cells with low photosynthetic capacity that are surprised with LL at lights-on may have lower induction than do cells that have increased their inventory of antenna complexes by the second LL day, and vice versa (Fig. 5).

In our controlled, reductionist experimental system, the light intensity remains constant during the light phase. In nature, a day may start off gloomy and turn out bright, or the fog may roll in after a sunny morning. Future work should interrogate Chlamydomonas’ competence to reacclimate to new light intensities at various times of the diurnal cycle and/or cell cycle. Given our results demonstrating the alga’s impressive flexibility, we suspect that Chlamydomonas has evolved to stay light on its feet and can adapt to just about any day.

## Materials and Methods

### Strains and Culture Conditions.

*C. reinhardtii* strain CC-5390 was grown in High Salt (HS) medium with a modified trace element solution aerated with filter-sterilized air in flat-panel turbidostat FMT 150 Photobioreactors (Photon Systems Instruments, Drásov, Czechia) as previously described (30, 31). Cultures were subjected to diurnal cycles of 12 h light (80% blue light, 20% red light) at 50 (LL) or 1,000 (HL) μmol photons m^–2^ s^–1^ and 28 °C followed by 12 h dark and 18 °C. The LL-acclimated population was first synchronized in diurnal ML (200 µmol photons m^–2^ s^–1^) for 1 wk and then acclimated to diurnal LL (50 µmol photons m^–2^ s^–1^) for > 1 wk. The HL-acclimated population was synchronized and acclimated in diurnal HL (1,000 µmol photons m^–2^ s^–1^) for > 1 wk. At the start of the experiment (–12, t = 0 h), culture density was set to OD_680_ = 0.4 and medium flow was stopped. Then, the LL-acclimated populations were subjected to diurnal HL conditions, and the HL-acclimated populations were subjected to diurnal LL conditions. After the first 26 h (–10), experimental cultures were diluted back to OD_680_ = 0.4 and medium flow was stopped again before the second day of the experiment. Once a photoacclimated population had been transitioned to a new light intensity and used for a 2-d experiment, it was discarded and replaced with a fresh, naïve culture. Experimental replicates refer to independent experiments performed on cultures grown in different bioreactors months apart from each other. All measurements were taken for at least three experimental replicates.

### Productivity Measurements.

Cell growth was assessed by continuously monitoring culture OD_680_, periodically sampling for cell number and diameter with a Z2 Coulter Particle Count and Size Analyzer (Beckman Coulter, CA), and periodically measuring NPOC using a TOC-L Shimadzu Total Organic Carbon Analyzer (Shimadzu, Kyoto, Japan). Photosynthetic activity was assessed by measuring F_v_/F_m_, NPQ, and steady-state Chl fluorescence emission spectra at 77 K. Cellular pigment content was measured by HPLC. Detailed methods for NPOC, Chl fluorescence, and pigment content measurements are available in *SI Appendix*, *Supporting Information Text*.

### Cellular Morphology.

Cultures were sampled for live-cell imaging by Airyscan microscopy of Chl fluorescence. Samples were kept on ice in the dark for <30 min prior to sample preparation and imaging. For TEM, cells were fixed in 2% glutaraldehyde in HS medium at 4 °C with rotatory agitation in the dark for >10 h. Cells were stained, dehydrated, embedded, sectioned, and imaged as previously described (30). FIJI image analysis software was used to analyze the height of thylakoid membrane stacks and the gray value profiles of the chloroplast stroma and the cytoplasm in the micrographs. More details on sample preparation, imaging, and quantitative image analysis can be found in *SI Appendix*, *Supporting Information Text*.

### RNA-Seq.

Total RNA was extracted at 19 timepoints using the ZymoBIOMICS RNA Mini Kit (Zymo, CA). Total RNA was subjected to poly(A) selection, RNA-Seq library construction, and sequencing on the Illumina Novaseq X Plus 10B platform by the University of California Los Angeles Technology Center for Genomics and Bioinformatics (UCLA, CA) using standard kits and protocols (Illumina Inc. CA). Reads were mapped to the *C. reinhardtii* reference genome assembly and annotations v6.1 (82).

8 of the 114 samples experienced changes in sample volume during sample shipment and were flagged by the sequencing facility for possible evaporation or cross-contamination between wells of the 96-well plate (*SI Appendix*, Fig. S11*A*). PCA of all samples showed that these samples were dissimilar from the other experimental replicates of the condition (*SI Appendix*, Fig. S11*B*). These 8 libraries were therefore discarded, leaving at least two experimental replicates for each treatment group. Only nucleus-encoded mRNAs that were detected with a maximum FPKM > 1 were analyzed (15,305 genes) (Dataset S2). Additional information about RNA-Seq methodology can be found in *SI Appendix*, *Supporting Information Text*. Raw RNA-Seq data have been deposited in National Center for Biotechnology Information Gene Expression Omnibus (NCBI GEO) under accession GSE307139 (83).

### TMT Proteomics.

Cultures were sampled for tandem mass tag (TMT) proteomics at 10 timepoints. Cells were washed in 10 mM Na-phosphate buffer (pH 7.0), and the washed cell suspensions were flash-frozen in liquid N_2_ and stored at –80 °C until further processing. Cells were lysed by bead-beating in 8 M urea. Proteins in the lysate were reduced, alkylated, and then digested with 1:50 (w/w) trypsin and 1:20 (w/w) Lys-C proteases. Further sample processing, TMT peptide labeling, LC-MS/MS data collection, and proteomics data processing were performed as previously described (30).

While 11,489 proteins were detected across the dataset, only proteins that were detected in at least two of the three experimental replicates at all timepoints in either population were analyzed (9,567 proteins total) (Dataset S2). Additional details about proteomics methodology can be found in *SI Appendix*, *Supporting Information Text*. Raw proteomics data have been deposited in Mass Spectrometry Interactive Virtual Environment (MassIVE) under accession MSV000098920 (84).

### Transcriptomic and Proteomic Data Analysis.

Gene-wise normalization was performed for each transition independently (LL to HL or HL to LL). PCA of the transcriptome and proteome was performed using the R package *PCAtools* (v2.16.0) on mRNAs with a minimum average FPKM > 1 (7,613 genes), and proteins with a maximum average MASIC that was over the limit of quantitation (MASIC > 12.6) (8,922 proteins in the LL-to-HL transition, 8,984 proteins in HL-to-LL transition). *k*-means clustering analysis was conducted using the R package *stats* (v4.4.0). First, mean transcript abundances (as FPKMs) and mean protein abundances (as MASIC values) were Z-score normalized across time in a given transition (LL to HL or HL to LL). Only genes whose expression was detected at both the mRNA and protein levels at all timepoints of the transition were included (8,821 genes in LL to HL, and 8,868 genes in HL to LL) (Dataset S2), and the kmeans function was applied with centers = 16 and iter.max = 1,000. Each cluster of genes was tested for GO term enrichment using the enricher function from the R package *clusterProfiler* (v4.12.0) with pvalueCutoff = 0.05 and pAdjustMethod = “BH.” Representative enriched GO terms are displayed. The full list of significantly enriched GO terms is available as Dataset S3.

Line plots of mean gene expression and the 95% CI across the experimental replicates were generated in the R package *ggplot2* (v3.5.1) using the mean_cl_boot function. Composite heatmaps of gene expression patterns were generated in the R package *ComplexHeatmaps* (v2.20.0) (85).

## Supplementary Material

Appendix 01 (PDF)

Dataset S01 (XLSX)

Dataset S02 (XLSX)

Dataset S03 (XLSX)

## Data Availability

Raw RNA-Seq data and raw proteomics data have been deposited in NCBI GEO (GSE307139) and MassIVE (MSV000098920), respectively (83, 84). Previously published data were used for this work. Physiological results for fully HL-acclimated Chlamydomonas populations and fully LL-acclimated Chlamydomonas populations shown for comparison with the data presented in this work are reproduced from Dupuis et al. (30) and are publicly available. Gene expression data for ML-acclimated Chlamydomonas populations shown for baseline comparison are reproduced from Strenkert et al. (31) (publicly available at NCBI Gene Expression Omnibus accession GSE112394). All other data are included in the manuscript and/or supporting information.
